# ^161^Terbium-Labeled Gold Nanoparticles as Nanoscale Brachytherapy Agents Against Breast Cancer

**DOI:** 10.3390/ma18020248

**Published:** 2025-01-08

**Authors:** Evangelia-Alexandra Salvanou, Adamantia Apostolopoulou, Stavros Xanthopoulos, Stuart Koelewijn, Philippe van Overeem, Gautier Laurent, Rana Bazzi, Franck Denat, Stéphane Roux, Penelope Bouziotis

**Affiliations:** 1 Radiochemical Studies Laboratory, Institute of Nuclear & Radiological Sciences & Technology, Energy & Safety, National Center for Scientific Research “Demokritos”, Patriarchou Grigoriou and 27 Neapoleos Street, 15341 Athens, Greece; a.apostolopoulou@rrp.demokritos.gr (A.A.); staxan@rrp.demokritos.gr (S.X.); bouzioti@rrp.demokritos.gr (P.B.); 2 Terthera b.v., Minervum 7070, 4817 ZK Breda, The Netherlands; stuart@terthera.com (S.K.); philippe@terthera.com (P.v.O.); 3 Laboratoire Chrono-Environnement, Université de Franche-Comté, CNRS, F-25000 Besançon, France; gautier.laurent0@gmail.com (G.L.); rana.bazzi@univ-fcomte.fr (R.B.); stephane.roux@univ-fcomte.fr (S.R.); 4 Institut de Chimie Moléculaire de l’Université de Bourgogne, UMR 6302, CNRS, Université de Bourgogne, F-21078 Dijon, France; franck.denat@u-bourgogne.fr

**Keywords:** Terbium-161, nanoscale brachytherapy, gold nanoparticles, radiolabeling, MTT, breast cancer, biodistribution, therapeutic efficacy

## Abstract

Due to their intriguing emission profile, Terbium-161 (^161^Tb) radiopharmaceuticals seem to bring significant advancement in theranostic applications to cancer treatment. The combination of ^161^Tb with nanoscale brachytherapy as an approach for cancer treatment is particularly advantageous and promising. Herein, we propose the application of a hybrid nanosystem comprising gold decorated (Au@TADOTAGA) iron oxide nanoflowers as a form of injectable nanobrachytherapy for the local treatment of breast cancer. More specifically, Au@TADOTAGA and NFAu@TADOTAGA NPs were efficiently radiolabeled with ^161^Tb, and their in vitro stability was assessed up to 21 d post-radiolabeling. Furthermore, their cytotoxic profile against 4T1 breast cancer cells was evaluated, and their ex vivo biodistribution characteristics were revealed after intratumoral injection in the same animal model. The enhanced retention at the tumor site urged us to evaluate the therapeutic effect of the [^161^Tb]Tb-NFAu@TADOTAGA nanosystem after intratumoral administration to 4T1-tumor-bearing mice, over a period of 24 days. Three different therapeutic protocols were performed in order to identify which therapeutic approach would offer the optimum results and identify the proposed nanosystem as a promising nanoscale brachytherapy agent.

## 1. Introduction

The standard of care for breast cancer treatment involves a multidisciplinary approach that typically includes surgery, chemotherapy, radiation therapy, hormone therapy, and targeted therapies. Surgery is often the first line of treatment, and following surgery, radiation therapy may be employed to eliminate any remaining cancer cells and reduce the risk of recurrence. Chemotherapy can be administered before surgery to shrink tumors or after to prevent metastasis. The goal in cancer treatment is to make a more personalized treatment plan that would be able to adapt to the needs of the patient according to the type and stage of cancer and overall health in order to optimize outcomes and manage potential side effects effectively.

Radiation therapy (RT) is a cornerstone treatment for cancer that employs high-energy radiation to destroy cancer cells and therefore shrink tumors. This therapeutic approach can be utilized as a curative treatment, as an enhancement for the effectiveness of other therapies like chemotherapy (neo-adjuvant radiotherapy), as a preventative for cancer recurrence post-surgery (adjuvant radiotherapy), or as a palliative agent in alleviating symptoms. Radiation therapy can be delivered through different methods: systemic RT, which involves administering radioactive substances via injections or oral intake; external beam radiation therapy (EBRT), which targets tumors from outside the body; and brachytherapy, where radioactive implants are placed inside or near the tumor [1]. Overall, RT is a widely used method, with approximately 60% of patients receiving it as part of their treatment regimen [2,3]. However, the drawback is that it can also affect healthy tissues, leading to side effects such as fatigue, skin irritation, and hair loss in the treated area.

Terbium radiopharmaceuticals, particularly those utilizing the radionuclide Terbium-161 (^161^Tb), are gaining attention in the field of nuclear medicine for their potential in targeted cancer therapy [4,5,6]. ^161^Tb is a promising radionuclide in the field of targeted radionuclide therapy, with interesting properties similar to another well-established radioisotope used in cancer treatment, Lutetium-177 (^177^Lu). ^161^Tb is a radiolanthanide with a half-life of 6.9 days that decays to stable Dysprosium-161 (^161^Dy) through beta^−^ (β^−^) emission. Furthermore, unlike ^177^Lu, ^161^Tb emits a considerable number of conversion and Auger electrons along with beta particles, which deliver localized radiation within cancer cells, enhancing the damaging of the DNA of tumors, especially in microscopic or single-cell metastases that are often missed by other treatments with^177^Lu radiopharmaceuticals. Studies have shown that ^161^Tb increases the delivered dose per unit of activity to tumor tissue by 40% compared to ^177^Lu, making it more effective in eliminating minimal residual disease [7,8,9]. Its decay process enables it as a theranostic isotope, as apart from the emission of beta particles with a mean energy (E_β_) of about 154 keV, it also emits gamma radiation, specifically at energies of 49 keV (17%) and 75 keV (10%), which are useful for imaging purposes, such as single-photon emission computed tomography (SPECT) imaging [4]. The similarities in the decay profile, which are comparable to those of ^177^Lu, allow for similar logistical considerations in its clinical application and translation, as protocols developed for ^177^Lu can be adapted for ^161^Tb while improving the overall therapeutic index. ^161^Tb can be linked to ligands that specifically target tumor cells, enhancing its uptake by cancerous tissues while minimizing exposure to healthy cells and side effects. Preclinical studies have demonstrated that ^161^Tb-based therapies outperform respective ^177^Lu-based therapies in various cancer cell lines and animal models [10,11,12,13,14]. This targeted approach not only increases the therapeutic index but also reduces potential side effects associated with radiation therapy. Currently, several clinical trials are underway to evaluate the potential, safety, and effectiveness of ^161^Tb-based radiopharmaceuticals for targets like neuroendocrine tumors and prostate cancer [15,16,17]. These trials aim to confirm its potential as a more effective treatment option for patients with resistant or recurrent cancers, highlighting its ability to deliver localized radiation doses that can significantly impact tumor growth and patient outcomes.

Nanoparticles (NPs) are revolutionizing cancer treatment by enhancing imaging and therapeutic capabilities. These nanoscale carriers offer several advantages over conventional therapies, including increased biocompatibility, reduced toxicity, and enhanced permeability and retention (EPR) effects [18,19]. By including targeting, chemotherapeutic, or radioactive agents in their structures, NPs can serve as targeted drug delivery systems directly to tumor sites, thus minimizing deleterious side effects [20,21,22,23,24]. Several nanomedicine products have already been used for cancer therapy with favorable clinical outcomes, while other types are currently being explored in clinical trials, showcasing their potential to transform cancer therapy into a more personalized and effective modality [25,26,27,28].

Nanoscale brachytherapy represents an innovative advancement in cancer treatment, utilizing radioactive NPs for targeted radiation therapy of solid tumors. Unlike traditional brachytherapy, which involves the implantation of larger radioactive seeds, nanoscale brachytherapy employs radiolabeled NPs that can be directly injected into tumors [29,30,31]. This method enhances the precision of radiation delivery, allowing for uniform dose distribution while minimizing damage to surrounding healthy tissues. Recent studies have demonstrated the efficacy of these nanoparticles in effectively arresting tumor growth in preclinical models [32,33,34,35,36]. The localized nature of nanoscale brachytherapy not only improves therapeutic outcomes but also reduces the invasiveness associated with conventional techniques, paving the way for its potential integration into standard cancer treatment protocols for unresectable solid tumors.

Under this scope, in the present study, our aim was to investigate the development and comparative study of a nanosystem comprising gold NPs (Au@TADOTAGA) radiolabeled with the Auger-emitter ^161^Tb as a nanoscale brachytherapy agent. Au@TADOTAGA nanoparticles radiolabeled with Actinium-225 ([^225^Ac]Ac-Au@TADOTAGA) behave as promising nanoscale brachytherapy agents [33]. Since the retention time is expected to be a key-parameter for nanoscale brachytherapy, we propose immobilization before radiolabeling ultrasmall gold nanoparticles (Au@TADOTAGA) onto larger iron oxide nanoflowers (IONF). After radiolabeling, free gold nanoparticles (Au@TADOTAGA) and immobilized gold nanoparticles (NFAu@TADOTAGA) were synthesized, radiolabeled, and evaluated in vitro and ex vivo in tumor-bearing mice. Ex vivo evaluation pinpointed the difference in the retention of the two examined nanostructures after their intratumoral administration, while a therapeutic efficacy study performed in the same animal model demonstrated their therapeutic potential.

## 2. Materials and Methods

Special radioprotective measures must be used when handling the ^161^Tb isotope to reduce the risk of harm. All radiolabeling operations and work with radiolabeled compounds were carried out in a radiochemistry facility that possesses the equipment, knowledge, and license required to carry out radioisotope experiments safely. Water for injection was purchased from DEMO S.A. (Krioneri Attiki, Greece). All other reagents and solvents used in these studies were obtained from commercial sources without further purification. Water was deionized to 18 MΩ⋅cm using an Easypure water filtration system (Barnstead International, Dubuque, IA, USA). Lab Companion CBS-350 heating shaker (JEIO TECH, Daejeon, Republic of Korea) and Digital Thermoblock TD 200 (Falc Instruments, Treviglio, Italy) were used. Materials used for the synthesis of gold nanoparticles were purchased from Sigma-Aldrich (St. Louis, MO, USA) and CheMatech, (Dijon, France).

^161^Tb was provided as [^161^Tb]TbCl_3_ by TerThera (Breda, The Netherlands). Radioactivity of [^161^Tb]TbCl_3_ and of the radiolabeled nanoparticles was measured using a dose calibrator (Capintec, Ramsey, NJ, USA). Citric acid 0.1 M was used as the mobile phase, and glass microfiber chromatography paper impregnated with silica gel (ITLC-SG, Agilent Technologies, Santa Clara, CA, USA) was the stationary phase for the radiolabeling and stability studies with a radio-TLC scanner (Scan-Ram, LabLogic, Sheffield, UK). The percentage of ^161^Tb incorporated onto the NPs was calculated as 100 × (counts at application point/total counts). Data collection and analysis were performed with Laura software v. 5.0.4.29. Human serum was acquired from Sigma-Aldrich (St. Louis, MO, USA).

The 4T1 murine cancer cell line was acquired from the cell bank of the Laboratory of Radiobiology, Institute of Nuclear & Radiological Sciences & Technology, Energy & Safety, NCSR “Demokritos”. The specific cell line mimics stage IV human breast cancer, and cells were verified to be free of mycoplasma contamination after staining with 4′,6-diamidine-2′-phenylindole dihydro-chloride (DAPI) and microscope observation.

Cells were grown in Roswell Park Memorial Institute 1640 Medium (RPMI, Biowest, Riverside, MO, USA), supplemented with 10% FBS, 100 U/mL of penicillin, 2 mM glutamine, and 100 μg/mL of streptomycin. Fetal bovine serum (FBS), 100 U/mL penicillin, 0.1 mg/mL streptomycin, trypsin-EDTA solution (0.25% trypsin/0.53 mM EDTA), phosphate-buffered saline (PBS) at a pH of 7.4, and dimethyl sulfoxide (DMSO) were obtained from ThermoFisher Scientific (Waltham, MA, USA). The MTT reagent (3-[4,5-dimethylthiazol-2-yl]-2,5-diphenyl-tetrazolium bromide) was purchased from Applichem (Darmstad, Germany). Cell viability was determined using a LabSystems Multiskan RC microplate reader (Thermo Fisher Scientific, Waltham, MA, USA).

For the animal experiments, SCID (severe combined immunodeficiency) mice of both genders were used. The mice were housed in individually ventilated cages (IVC) with constant temperature (22 ± 2 °C) and humidity (45–50%) and a 12 h light/dark cycle, with free access to food and water. Animals were obtained from the breeding facilities of the Institute of Biosciences and Applications, NCSR “Demokritos”. The experimental animal facility is registered according to the Greek Presidential Decree 56/2013 (Reg. Number: EL 25 BIO 022) in accordance with European Directive 2010/63, which is in accordance with national legislation regarding the protection of animals used for scientific purposes. All applicable national guidelines for the care and use of animals were followed. The study protocol was approved by the Department of Agriculture and Veterinary Service of the Prefecture of Athens. These studies have been further approved by our institutional ethics committee, and the procedures followed are in accordance with institutional guidelines.

Breast cancer xenografts were developed for the biodistribution and therapeutic efficacy studies. Isoflurane (1000 mg/g, Iso-Vet, Chanelle Pharma, Loughrea, Ireland) inhalation was used to sacrifice the animals. The radioactivity of samples and syringes was measured using a dose calibrator (Capintec, Ramsey, NJ, USA). An automatic gamma counter (Cobra II, Canberra, Packard, Schwadorf, Austria) was used to measure the radioactivity of each organ and blood sample in ex vivo biodistribution studies.

### 2.1. Synthesis

#### 2.1.1. Synthesis of Macrocyclic Chelator-Coated Gold Nanoparticles

The protocol published by Brust et al. for synthesizing gold nanoparticles is well suited for yielding ultrasmall gold nanoparticles coated with different types of ligand and in particular with hydrophilic molecules [37].

The synthesis of gold nanoparticles, coated with the polyaminocarboxylate chelator TADOTAGA, was performed by mixing under stirring HAuCl_4_ 3H_2_O (50 mg, 1.22 × 10^–4^ mol in 20 mL of methanol) with an aqueous solution of TADOTAGA (86 mg, 1.22 × 10^–4^ mol in 10 mL of water) in a 250 mL round-bottom flask. A color change from yellow to orange was observed. After five minutes, NaBH_4_ (48 mg, 12.7 × 10^–4^ mol in 3 mL of water) was introduced into the flask containing the mixture under vigorous stirring at room temperature (RT). Instantaneously, the orange solution became black. The suspension which was stirred for 1 h was purified by dialysis using a 6000–8000 molecular weight cut-off membrane.

#### 2.1.2. Synthesis of Maghemite Nanoflowers Decorated with Gold Nanoparticles

The maghemite nanoflowers decorated with gold nanoparticles (NFAu@TADOTAGA) were produced by grafting dopamine-modified Au@TADOTAGA nanoparticles (Au@TADOTAGAd) onto the iron oxide (maghemite γ-Fe_2_O_3_) nanoflowers (NF).

As demonstrated by Hugounenq et al., the synthesis of NF requires the use of a mixture of polyol solvents (diethyleneglycol (DEG) and N-methyldiethanolamine (NMDEA)) [38]. After 1 h stirring, a solution of DEG containing FeCl_3_ 6H_2_O (2.164 g; 8 mmol) and FeCl_2_·4H_2_O (0.796 g; 4 mmol) was poured with N-methyldiethanolamine (NMDEA, 75 mL) and stirred again for one hour. Freshly prepared solution of polyols (40 mL DEG and 40 mL NMDEA) containing NaOH (1.42 g; 35.6 mmol) was added to the solution of iron chlorides. The resulting mixture was stirred for 3 h. Then, a temperature ramp (2 °C·min^−1^) is applied until 220 °C. The solution stirring is maintained for 4 h at 220 °C and during the cooling step in ambient air to room temperature. The mixture of polyols and the solvent used for removing the impurities (mixture of ethanol and ethyl acetate (1:1, *v*/*v*)) were removed using a magnet owing to the magnetic properties of NF. A treatment with 10% nitric acid is then carried out for eliminating the iron hydroxides. The last step of the NF synthesis consists of the complete oxidation of the nanoparticles. After the addition of an aqueous solution of iron(III) nitrate (Fe(NO_3_)_3_·9H_2_O) (2 g, 4.951 × 10^−3^ mol in water (20 mL)), the NF suspension was heated to 80 °C for 45min. After another treatment with 10% nitric acid, the particles were washed twice with acetone and diethyl ether and redispersed in water. The resulting aqueous suspension of maghemite nanoflowers exhibits colloidal stability in acid or basic conditions with a point of zero charge near pH 7.3.

*Chelator-coated gold nanoparticles functionalized with dopamine (Au@TADOTAGAd).*

1-Ethyl-3-(3-dimethylaminopropyl)carbodiimide (EDC, 0.207g; 1.08 × 10^−3^ mol) and N-hydroxysuccinimide (NHS, 0.247 g; 2.150 × 10^−3^ mol) in water (3 mL) and a suspension of Au@TADOTAGA gold nanoparticles (6 mL, 10 g_Au_/L) were mixed together at pH 6 and stirred for 90 min. Prior to the addition of dopamine (9.45 × 10^−3^ g; 2.25 × 10^−5^ mol in water (4 mL)), pH of the suspension is fixed at pH 7.5. After the addition of dopamine, the suspension was stirred overnight. Impurities were removed from the suspension of gold nanoparticles by dialysis against water (MWCO: 6–8 kDa) for 12 h. Water bath was changed three times every 3 h.

*Synthesis of maghemite nanoflowers decorated with gold nanoparticles (NFAu@TADOTAGA).*

The immobilization of the gold nanoparticles onto NF was achieved by mixing the suspension of Au@TADOTAGAd in the NF suspension (6 mL; 35 g Fe/L). The aqueous suspension of gold nanoparticles and maghemite nanoflowers (pH 5.5) was heated at 50 °C for 24 h. Afterwards, the suspension was successively washed with ultrapure water, acetone, and diethyl ether until the supernatant was clear. An aqueous suspension of NFAu@TADOTAGA with an iron concentration of 35 g·L^−1^ was prepared from the powder collected after purification.

#### 2.1.3. Characterization of Au@TADOTAGA and NFAu@TADOTAGA Gold Nanoflowers

The hydrodynamic diameter and the zeta potential of nanoparticles were measured using Zetasizer from Malvern Instrument (Malvern, UK). The measurements of hydrodynamic diameter were performed with aqueous suspensions at a concentration in gold of 4 × 10^−4^ M. The measurements of zeta-potential were performed with aqueous suspensions (pH 7.4) at a concentration in gold of 4 × 10^−4^ M and in NaCl (0.01 M).

Detailed morphological information about the samples was obtained by transmission electron microscopy (TEM) experiments (JEOL 2010 microscope (JEOL, Tokyo, Japan) operating at 200 kV). The observation of the nanostructures by TEM required the preparation of grids (carbon film on Cu 300 square mesh, ultra-thin coating, Delta Microscopies, Mauressac, France), which consisted of the deposition of a drop of a diluted aqueous suspension of Au@TADOTAGA nanoparticles, NF, or NFAu@TADOTAGA. After drying in air at RT in clean environment, the grids were ready for TEM.

### 2.2. Radiolabeling

^161^Tb was acquired in the form of [^161^Tb]TbCl_3_ in aqueous 0.05 M HCl solution. Radiolabeling was achieved for both types of NPs (Au@TADOTAGA and NFAu@TADOTAGA) via the TADOTAGA chelator. The NP suspension was added to trace-free sodium acetate buffer (pH of 5.5) and incubated with [^161^Tb]TbCl_3_ for 1 h at 75 °C and 850 rpm.

For the determination of the radiochemical yield, ITLC-SG (citric acid, 0.1 M) was used, where [^161^Tb]Tb-NPs stayed at the application point (Rf = 0.0–0.2) and unbound ^161^Tb^3+^ was found at the solvent front (Rf = 0.8–1.0).

In vitro stability of the [^161^Tb]Tb-NPs was assessed at RT, phosphate buffer saline (PBS), and human serum. In particular, radiolabeled NPs were added to PBS or serum at a ratio 1:10 and incubated at 37 °C up to 21 d post-radiolabeling. Analysis was performed with ITLC-SG (0.1 M citric acid). All experiments were performed in triplicate from three independent radiolabeling procedures.

### 2.3. In Vitro Cytotoxicity

The 3-(4,5-dimethylthiazol-2-yl)-2,5-diphenyltetrazolium bromide (MTT) colorimetric assay was used to assess the unlabeled and ^161^Tb-labeled NPs’ in vitro cytotoxicity against the 4T1 triple-negative breast cancer cell line up to 72 h. Briefly, cells were seeded in 96-well plates (8 × 10^3^, 6 × 10^3^, and 4 × 10^3^ cells/well for the 24, 48, and 72 h incubation times, respectively) and grown overnight at 37 °C in a 5% CO_2_ incubator. The concentrations of non-radioactive NPs investigated ranged from 0.625 to 20 μg_Au_/mL and 0.125 to 4 MBq/mL for the radioactive NPs, which corresponds to the same concentration of non-radiolabeled NPs. In all cases, the final volume in each well was 100 μL at all examined concentrations. The medium was then removed and replaced with 100 μL of MTT solution (1 mg/mL). After a 4 h incubation, the solution was aspirated, formazan crystals were solubilized in 100 μL of isopropanol, and absorbance was recorded at 540 nm. The results were expressed as % cell viability = (mean optical density (OD) of treated cells/mean OD of untreated cells) × 100. Each assay was repeated three times.

### 2.4. Ex Vivo Biodistribution

All animal studies were performed on mice with severe combined immunodeficiency (NOD/SCID), 6–8 weeks old. Each mouse was subcutaneously inoculated in the region under the left front limb with 4T1cancer cells (5 × 10^6^ cells per mouse). Experimentation was initiated once the tumor was of a palpable size (~200 mm^3^).

Since the objective was the development of a nanoscale brachytherapy agent, administration of the ^161^Tb-labeled radioconjugates was performed intratumorally (i.t.) with 1 MBq/mouse for both [^161^Tb]Tb-Au@TADOTAGA and [^161^Tb]Tb-NFAu@TADOTAGA. Four animals per time point were euthanized at 1, 2, and 7 d post injection in a chamber saturated with isofluorane vapors. The organs and tissues of interest (i.e., heart, liver, spleen, lungs, kidneys, stomach, intestines, pancreas, and sample of bones and muscle), along with the tumor, were excised, weighed, and measured in an automatic γ-counter. Samples of blood and urine were also collected and measured. Accumulation of the radiolabeled NPs in organs and tissues at each time point was expressed as the mean percentage of injected activity per gram ± standard deviation (% IA/g ± SD), using an appropriate sample as a standard.

### 2.5. Therapeutic Efficacy

Therapeutic efficacy studies were performed in NOD/SCID mice bearing subcutaneous breast cancer tumors when the tumor reached a volume of about 150 mm^3^. Mice were randomly divided into four groups (*n* = 4 mice per group) and received intratumorally either normal saline (control group) or ^161^Tb-labeled NFAu@TADOTAGA. The groups injected with the radioactive compounds included the animals treated with one bolus injection of 5 MBq of [^161^Tb]Tb-NFAu@TADOTAGA, two injections with 5 MBq each of [^161^Tb]Tb-Au@TADOTAGA, and one bolus injection of 10 MBq of [^161^Tb]Tb-NFAu@TADOTAGA. Mice were monitored by measuring their body mass and potential signs of pain or unease three times a week. Tumor volume was monitored for 24 days using calipers and was calculated using the formula (length × width^2^)/2. The tumor growth index (TGI) for all animal groups was calculated by dividing the tumor volume of each day of measurement by the initial tumor volume at the initiation of the experiment (day 0), just before the intratumoral injection of ^161^Tb-labeled complexes. TGI was plotted vs. treatment time post injection.

### 2.6. Statistical Analysis

The data are presented as means ± standard deviations (SDs). For the cytotoxity and biodistribution results, data were compared using an unpaired *t*-test with a significance level of *p* < 0.05 by Microsoft Excel. Asterisks indicate the statistical significance of the difference between the results (* *p* < 0.05, ** *p* < 0.01, *** *p* < 0.001, **** *p*< 0.0001), while absence of asterisks indicates a non-significant statistical difference.

## 3. Results and Discussion

### 3.1. Synthesis

Au@TADOTAGA NPs are composed of an ultrasmall gold core (2–3 nm) coated with macrocyclic chelators (TADOTAGA). TADOTAGA was obtained through the modification of DOTAGA (macrocyclic polyaminocarboxylate) with thioctic acid (TA). DOTAGA is well known for its ability to form stable complexes with lanthanide(III) ions (such as gadolinium, europium, terbium), while TA, which is characterized by an endocyclic disulfide bond, exhibits a high affinity for gold surface [39,40,41]. The conjugation of DOTAGA with TA confers to TADOTAGA a capacity both to strongly adsorb onto the gold core and to immobilize ions of interest for imaging and for therapy. In previous studies, we demonstrated that Au@TADOTAGA NPs behave as positive contrast agents for magnetic resonance imaging (MRI) when Au@TADOTAGA NPs are labeled by gadolinium ions (Au@TADOTAGA(Gd)); as radiotracers after radiolabeling with indium-111 ions, which form stable complexes with TADOTAGA (Au@TADOTAGA(In-111)); and as radioenhancers for external beam radiotherapy because of the presence of elemental gold in the NPs. Au@TADOTAGA(Gd) nanoparticles exhibit, therefore, a high potential for radiotherapy guided by MRI [42]. DOTAGA is also able to form stable complexes with radioactive actinium-225 (^225^Ac). Since Ac-225 is an α-emitter with a high potential for internal radiation therapy, the immobilization of this radioisotope confers to [^225^Ac]Ac-Au@TADOTAGA nanoparticle features suited for nanobrachytherapy. The intratumoral injection of [^225^Ac]Ac-Au@TADOTAGA to mice bearing tumors led to an efficient control of tumor growth [33]. In order to increase the tumor retention time, the immobilization of these ultrasmall gold nanoparticles onto IONF has been envisaged. IONF are composed of small crystalline maghemite nanoparticles assembled in a flower-shaped structure. For achieving such an immobilization, Au@TADOTAGA nanoparticles are modified with dopamine using a protocol developed for grafting Au@DTDTPA gold nanoparticles onto IONF [43]. The presence of amine in dopamine and of a large number of COOH in the organic shell of the gold nanoparticles renders possible the functionalization of the gold nanoparticles by dopamine via the formation of an amide linkage. As a result, gold nanoparticles are decorated with catechol moieties, which exhibit a great affinity for the iron oxide surface.

**Figure 1:**
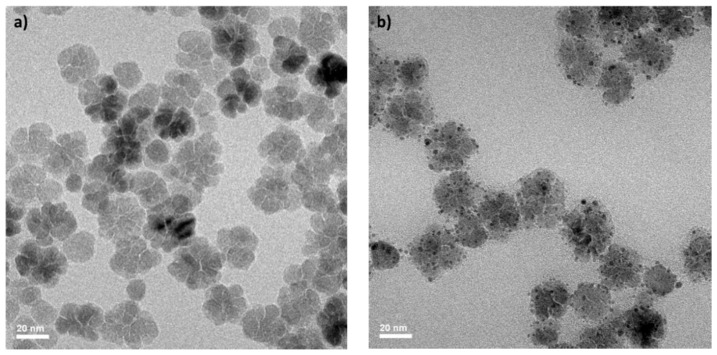
TEM images of (**a**) NF; (**b**) NFAu@TADOTAGA. Scale bar 20 nm.

**Figure 2:**
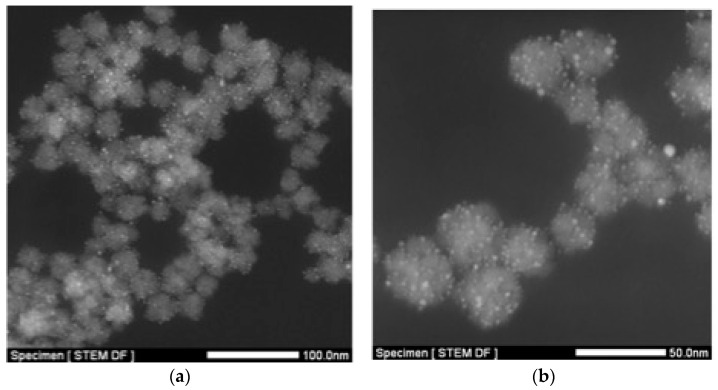
STEM-HAADF images of NFAu@TADOTAGA at different magnification (**a**) scale bar 100 nm; (**b**) 50 nm.

Iron oxide nanoflowers decorated with ultrasmall gold nanoparticles (NFAu@TADOTAGA) were obtained with a size between 27 and 33 nm and a hydrodynamic diameter of approximately 72 nm by magnetic sorting of an aqueous suspension prepared by mixing IONF and Au@TADOTAGA NPs at 50 °C under stirring for 24 h (Table 1). The presence of the gold nanoparticles onto the nanoflowers was not easy to observe on TEM images and was confirmed by STEM-HAADF (Figure 1 and Figure 2). On the micrographs, bright dots onto grey structures correspond to the gold nanoparticles onto the nanoflowers. It must also be pointed out that the presence of Au@TADOTAGA NPs onto IONF considerably improves the colloidal stability of the nanoflowers at physiological pH. The improvement of the colloidal stability observed for NFAu@TADOTAGA in comparison to IONF stems from the high hydrophilic character of the TADOTAGA shell onto the gold core and from the strongly negative zeta potential of Au@TADOTAGA nanoparticles.

### 3.2. Radiolabeling

**Figure 3:**
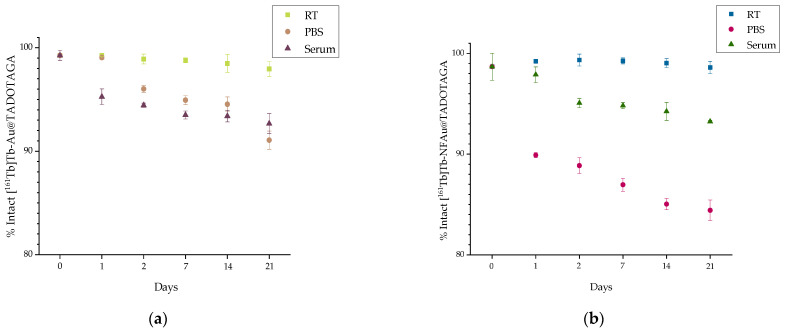
Radiochemical stability data of (**a**) [^161^Tb]Tb-Au@TADOTAGA and (**b**) [^161^Tb]Tb-NFAu@TADOTAGA at room temperature (RT), phosphate buffer saline (PBS) at 37 °C, and human serum at 37 °C (x axis not in scale).

Radiolabeling of both Au@TADOTAGA and NFAu@TADOTAGA was facile and robust. More specifically, 1 h at 75 °C and 850 rpm was enough to accomplish radiolabeling yields > 95% for both complexes without need for further purification (99.26 ± 0.50% for the [^161^Tb]Tb-Au@TADOTAGA and 98.68 ± 1.34% for the [^161^Tb]Tb-NFAu@TADOTAGA). An assessment of the radiolabeling and in vitro stability of the radiolabeled products took place with radio-TLC. These radionanoconjugates showed excellent stability at room temperature (97.95 ± 0.71% and 98.60 ± 0.61%) up to 21 d post radiolabeling, as depicted in Figure 3.

The in vitro stability of the ^161^Tb-labeled NPs in both the serum and PBS was good, with the minimum percentages recorded for serum stability as 92.67 ± 0.97% and 93.23 ± 0.12% for the [^161^Tb]Tb-Au@TADOTAGA and [^161^Tb]Tb-NFAu@TADOTAGA, respectively.

### 3.3. In Vitro Cytotoxicity

Both non-radiolabeled (“cold”) and ^161^Tb-labeled NPs were investigated concerning their cytotoxic effect up to 72 h against the 4T1 breast cancer cells. Cells were incubated with the same concentration of NPs in all cases.

#### 3.3.1. Toxicity of “Cold” Au@TADOTAGA and NFAu@TADOTAGA

**Figure 4:**
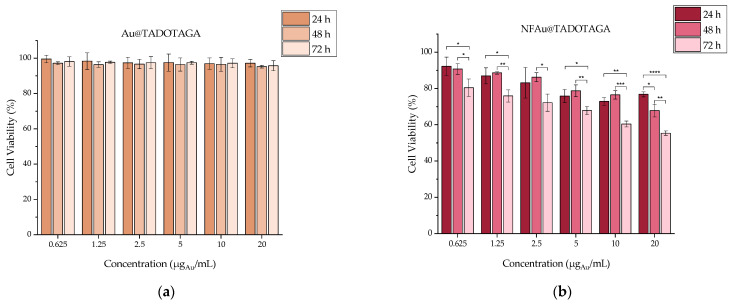
Cell viability assessed by MTT assay of (**a**) Au@TADOTAGA and (**b**) NFAu@TADOTAGA against the 4T1 cell line up to 72 h (* *p* < 0.05, ** *p* < 0.01, *** *p* < 0.001, **** *p* < 0.0001); absence of asterisks indicates a non-significant statistical difference). Values represent the mean ± SD (n = 3).

The cytotoxic effect of Au@TADOTAGA and NFAu@TADOTAGA is summarized in Figure 4. It is obvious that Au@TADOTAGA show almost no toxicity against 4T1 cells, as high cell viability percentages (>90%) are observed in all concentrations and incubation times. On the contrary, NFAu@TADOTAGA show a dose- and time-dependent toxicity with the highest effect being noted at 72 h and 20 μg_Au_/mL. Nonetheless, cell viability is above 50% in all examined conditions.

In our previous study, Au@TADOTAGA NPs have demonstrated a higher toxicity against glioblastoma U87MG cells at 20 μg_Au_/mL at 24 and 48 h incubation [33]. Also, nanostructures similar to NFAu@TADOTAGA have been examined before regarding their cytotoxic effect on various cell lines (EGI-1, hTERTHSC, HUVEC, and RAW 264.7) [43]. More specifically, in the case of HUVEC and hTERT cells, 24 h incubation with 200 and 500 μg/mL proved to reduce viability by ~50%. On the contrary, IONF as demonstrated in the same study or against LLC and CULA lung cancer cell lines demonstrated no major toxicity [44]. Also, Christou et al. have also synthesized iron–gold nanoflowers and have evaluated the cytotoxicity in HFF-1 cells (up to 50 µg/mL Fe + Au). The results confirmed that the nanoflowers reported no significant cytotoxicity [45].

#### 3.3.2. Toxicity of ^161^Tb-Labeled Au@TADOTAGA and NFAu@TADOTAGA

**Figure 5:**
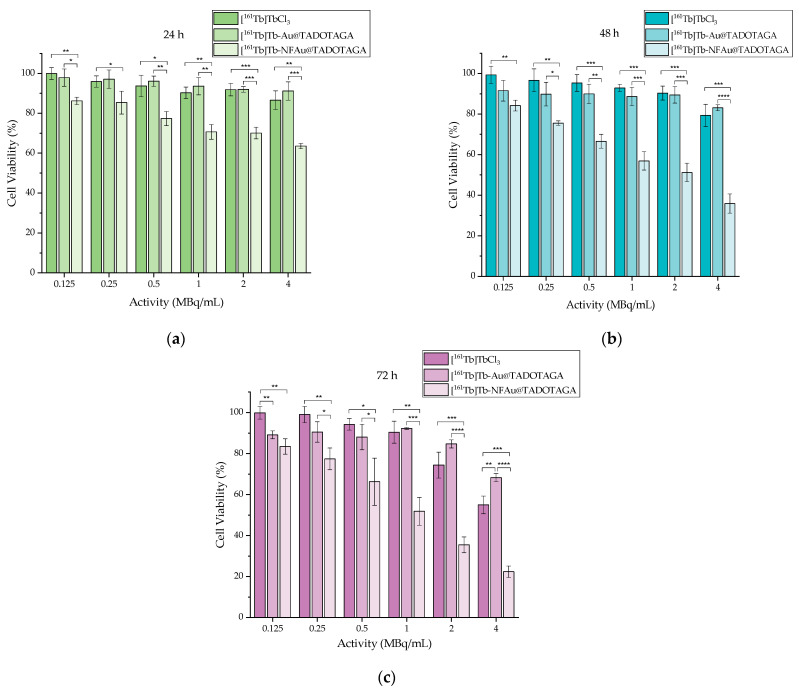
Cell viability assessed by an MTT assay of [^161^Tb]TbCl_3_, [^161^Tb]Tb-Au@TADOTAGA, and [^161^Tb]Tb-NFAu@TADOTAGA against the 4T1 cell line after (**a**) 24 h; (**b**) 48 h; (**c**) 72 h (* *p* < 0.05, ** *p* < 0.01, *** *p* < 0.001, **** *p* < 0.0001; absence of asterisks indicates a non-significant statistical difference). Values represent the mean ± SD (n = 3).

Figure 5a–c summarize the results of the cell toxicity induced after treatment for 24, 48, and 72 h with [^161^Tb]TbCl_3_, [^161^Tb]Tb-Au@TADOTAGA, and [^161^Tb]Tb- NFAu@TADOTAGA.

As can be seen in the above Figure 5, 72 h of the incubation of 4T1 cells with 4 MBq [^161^Tb]Tb-Au@TADOTAGA causes a relatively low decrease in cell viability (~32%), while [^161^Tb]TbCl_3_ and [^161^Tb]Tb-NFAu@TADOTAGA cause approximately 45 and 78% reduction, respectively.

The cytotoxicity of the Au@TADOTAGA NPs after radiolabeling with the alpha emitter, ^225^Ac, showed a significant reduction in cell viability of U87MG cells when compared to the respective cytotoxicity of the same NPs with ^161^Tb against 4T1 cells [33]. After 24 and 48 h incubation at the highest examined radioactivity of 2 kBq/mL of [^225^Ac]Ac-Au@TADOTAGA, a significantly higher cell cytotoxicity was observed. More specifically, cell viability at both time points was approximately ~20% and lower, whereas in the present study, the viability of 4T1 cells after 24 and 48 h incubation with 4 MBq/mL[^161^Tb]Tb-Au@TADOTAGA was found to be 91.18 ± 4.57% and 83.08 ± 1.48%. The different toxicity results cannot be attributed to the NPs’ concentrations, as they are the same in both studies (20 μg_Au_/mL), but they can be due to the different cell lines used (U87MG and 4T1) and more importantly to the different emitter and radioactivity conjugated to our nanosystem (^225^Ac vs. ^161^Tb). Borgna et al. have assessed the potential cytotoxicity of ^161^Tb-labeled SST analogues (0.01–1.00 MBq), and the results indicated that in all cases, the cell viability was very low [11]. Hence, regarding our cytotoxicity experiments, we were very interested in examining not only the ^161^Tb-radiolabeled nanostructures but also ^161^TbCl_3_ at the same activity range.

### 3.4. Ex Vivo Biodistribution

**Figure 6:**
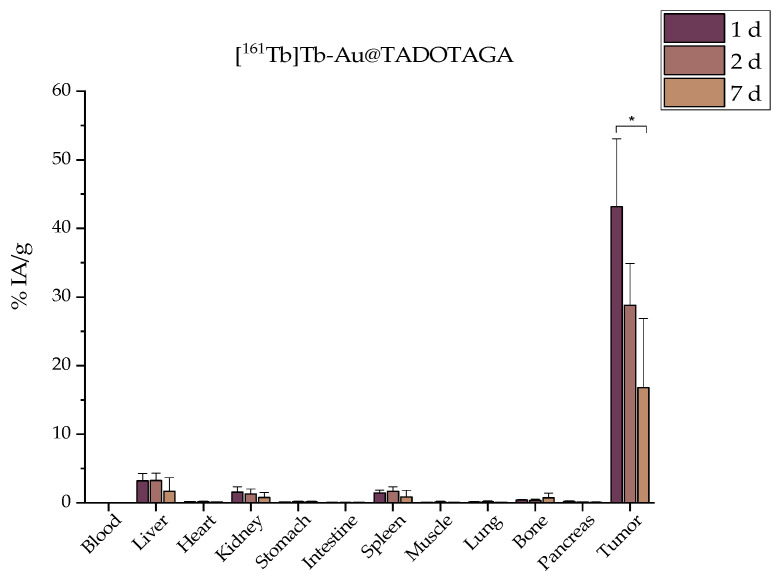
Ex vivo biodistribution after i.t. administration of [^161^Tb]Tb-Au@TADOTAGA in 4T1 xenografts expressed as % IA/g (n = 4, * *p* < 0.05; absence of asterisks indicates a non-significant statistical difference).

**Figure 7:**
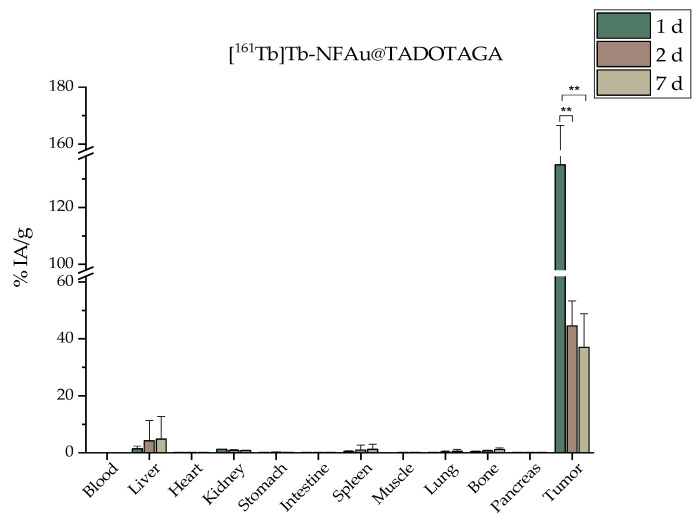
Ex vivo biodistribution after i.t. administration of [^161^Tb]Tb-NFAu@TADOTAGA in 4T1 xenografts expressed as % IA/g (n = 4, ** *p* < 0.01; absence of asterisks indicates a non-significant statistical difference).).

The biodistribution profile of the radiolabeled conjugates was assessed at 1, 2, and 7 days after intratumoral administration (Figure 6 and Figure 7). In both [^161^Tb]Tb-Au@TADOTAGA and [^161^Tb]Tb-NFAu@TADOTAGA, the injected amount was 40 μg_Au_, and the injected activity was 1 MBq per mouse.

The effective retention of radiolabeled NPs in tumors not only optimizes radiation exposure to cancerous cells but also represents a promising strategy for enhancing the overall success of brachytherapy treatments. In our case, it is clearly demonstrated that [^161^Tb]Tb-NFAu@TADOTAGA show a significantly more increased uptake at 1 d p.i. (134.96 ± 31.59 vs. 43.16 ± 9.93% IA/g, ** *p* < 0.01). Results at 7 d p.i. demonstrate that 16.80 ± 10.05% IA/g of the injected [^161^Tb]Tb-Au@TADOTAGA NPs is still at the tumor site, while the respective percentage for the [^161^Tb]Tb-NFAu@TADOTAGA is much higher (37.03 ± 11.81% IA/g). It is also important to mention that as seen in the biodistribution results, bone and liver uptake is negligible even one week after administration, which proves the robust and intact labeling of our nanostructures [46]. Furthermore, all major organs noted less than 5% IA/g in the case of [^161^Tb]Tb-Au@TADOTAGA and 15% IA/g in the case of [^161^Tb]Tb-NFAu@TADOTAGA at all time points. We have previously reported the biodistribution profile of ^225^Ac-labeled Au@TADOTAGA NPs, and the results are comparable to the ^161^Tb-labeled NPs both at 24 h and 5 d post-intratumoral administration [33]. Non-targeted iron oxide nanoflowers radiolabeled with ^177^Lu have demonstrated lower retention percentages at the injection site after 7 d (12.44 ± 2.71 vs. 37.03 ± 11.81% IA/g) [32]. Regarding the tumor/organ ratios presented in Table 2 and Table 3, all presented ratios are more pronounced for [^161^Tb]Tb-NFAu@TADOTAGA, especially at 1 d p.i. This trend is also observed up to 7 d p.i., with the exception of the tumor/liver ratio of [^161^Tb]Tb-Au@TADOTAGA NPs, which is slightly more prominent. These data demonstrate the superiority of [^161^Tb]Tb-NFAu@TADOTAGA in the preclinical setting and justify the use of this nanostructure for the therapeutic efficacy study that followed.

### 3.5. Therapeutic Efficacy Study

**Figure 8:**
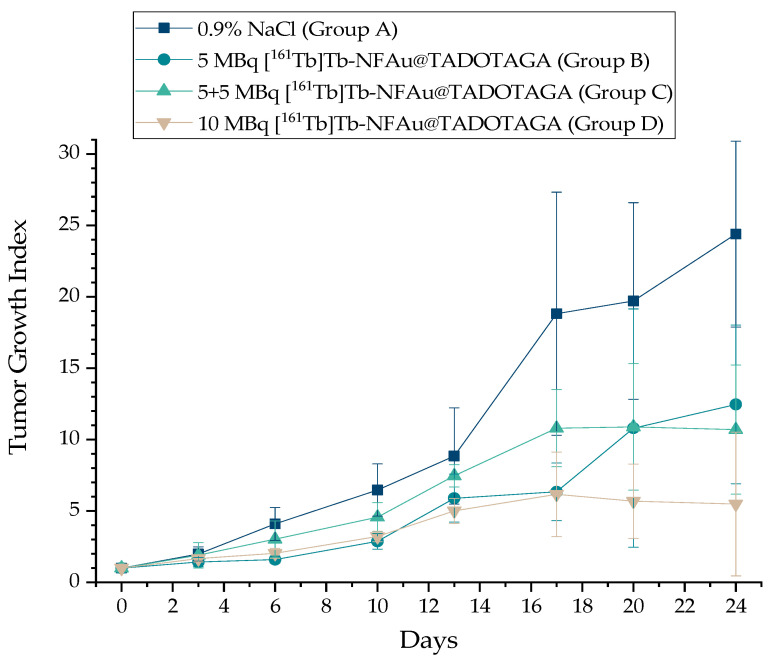
The therapeutic effect after the intratumoral administration of Group A: 0.9% NaCl (control group); Group B: a single dose of 5 MBq of [^161^Tb]Tb-NFAu@TADOTAGA; Group C: two doses of 5 MBq of [^161^Tb]Tb-NFAu@TADOTAGA; and Group D: a bolus dose of 10 MBq of [^161^Tb]Tb-NFAu@TADOTAGA in 4T1 tumor-bearing mice. Values represent the mean ± SD (n = 4 mice per group).

The localized delivery of radiation from the retained nanoparticles at the tumor site is expected to result in a higher dose concentration while sparing surrounding healthy tissues, thus minimizing side effects and improving therapeutic efficacy. Therefore, the therapeutic efficacy study was assessed only for the ^161^Tb-labeled NFAu@TADOTAGA, where the highest tumor retention was observed in the ex vivo biodistribution studies (37.03 ± 11.81 vs. 16.80 ± 10.05% IA/g at 7 d p.i.). Three different therapeutic protocols were performed in order to evaluate the variation in response among a bolus dose of 5 MBq, two doses of 5 MBq, and a bolus dose of 10 MBq. In the case of the double dose, the second administration was performed one half-life (7 d) after the first dose. The results of all groups are demonstrated in Figure 8.

The treatment of all groups was initiated simultaneously, and mice that were assigned to Group C for the second therapeutic dose of 5 MBq were the mice with the larger tumor volumes. This might justify the increasing trend of this group, which, however, seems more promising, as it starts delaying tumor growth more in comparison to Group B after day 20. The experiment was terminated when mice in the control group (Group A) met the endpoint criteria. Figure 8 indicates a dose-dependent therapeutic effect when compared to the control group, while none of the other animals in the treatment groups reached early-termination criteria. To our knowledge, this is the first study that investigates a ^161^Tb-radioconjugate after intratumoral administration. Therefore, a comparison with other studies reporting the therapeutic effect after the intratumoral administration of nanoconjugates radiolabeled with different radioisotopes is provided. The TGI of [^225^Ac]Ac-Au@TADOTAGA NPs, intratumorally injected with a total activity of 15 kBq in U87MG tumor-bearing mice, has shown a ~4-fold decrease at 22 days p.i. when compared to the control group, which is comparable to the one reported in the present work, which is ~4.4 [33]. Also, the therapeutic effect of i.t.-injected gold NPs radiolabeled with ^198^Au was monitored by Chanda et al. over a period of 30 d and was 5-fold smaller than the control group [47].

## 4. Conclusions

The hybrid nanoparticles synthesized and investigated in the present study proved to be stable carriers of the Auger-emitting radioisotope ^161^Tb. Cell toxicity was proved to increase depending on the dose and incubation time. Tumor retention after intratumoral administration in 4T1-tumor-bearing mice was clearly more augmented in the case of [^161^Tb]Tb-NFAu@TADOTAGA when compared to the respective case of [^161^Tb]Tb-Au@TADOTAGA. Therapeutic efficacy studies suggest that a higher dose of [^161^Tb]Tb-NFAu@TADOTAGA was responsible for tumor regression. After an assessment of the therapeutic efficacy of [^161^Tb]Tb-NFAu@TADOTAGA, the acquired promising results position it as a potentially transformative agent in the field of radionuclide therapy for cancer. The development of a nanosystem, as the one described in the present study, which combines the advantages of both gold and iron oxide nanoparticles but at the same time can be robustly radiolabeled with this particularly interesting radioisotope, could provide a radiopharmaceutical with high theranostic potential. This potential is not limited to the therapeutic and imaging capabilities of the Auger emitter ^161^Tb, but it can also offer the opportunity for imaging with magnetic resonance imaging (MRI) or computed tomography (CT) due to the iron and gold materials, respectively. Furthermore, magnetic hyperthermia due to the iron oxide nanoflowers and photothermal therapy due to the gold nanoparticles could potentially offer a synergistic therapeutic effect. To our knowledge, there is no other ^161^Tb nanobrachytherapy agent mentioned in the literature. Therefore, further experiments with different animal models and for a longer period of time will further elucidate its effectiveness and pave the way for its application as a nanobrachytherapy agent in other cancer types.

## Figures and Tables

**Table 1:** Core size, hydrodynamic diameter, and zeta-potential at pH 7.4 of Au@TADOTAGA nanoparticles, IONF, and NFAu@TADOTAGA.

	Au@TADOTAGA	IONF	NFAu@TADOTAGA
Core size (nm)	2.40 ± 0.60	27.1 ± 2.4	30.0 ± 3.0
Hydrodynamic diameter (nm)	8.30 ± 2.10	36.9 ± 2.0	71.5 ± 5.0
Zeta-potential (mV)	−25	−7.5	−35

**Table 2:** Tumor/non-target organ ratios for liver, kidneys, muscle, and bone for [^161^Tb]Tb-Au@TADOTAGA at all time points examined (1, 2, and 7 d).

	1 Day	2 Days	7 Days
Tumor/Liver	13.47	8.85	10.09
Tumor/Kidneys	27.71	22.30	21.58
Tumor/Muscle	692.39	256.83	483.21
Tumor/Bone	108.63	83.45	23.11

**Table 3:** Tumor/non-target organ ratios for liver, kidneys, muscle, and bone for [^161^Tb]Tb-NFAu@TADOTAGA at all time points examined (1, 2, and 7 d).

	1 Day	2 Days	7 Days
Tumor/Liver	95.62	10.60	7.75
Tumor/Kidneys	108.26	51.65	47.87
Tumor/Muscle	3059.71	420.82	563.32
Tumor/Bone	309.18	64.66	32.89

## Data Availability

The data presented in this study are available on request from the corresponding author. The data are not publicly available due to IP issues.
